# *RCAN1* Is an Important Mediator of Glucocorticoid-Induced Apoptosis in Human Leukemic Cells

**DOI:** 10.1371/journal.pone.0049926

**Published:** 2012-11-21

**Authors:** Kazuaki Nagao, Yujiro Iwai, Toshiyuki Miyashita

**Affiliations:** Department of Molecular Genetics, Graduate School of Medical Sciences, Kitasato University, Sagamihara, Japan; Emory University, United States of America

## Abstract

Glucocorticoid (GC) is a major therapeutic agent for the treatment of leukemia because of its ability to induce apoptosis in lymphoid cells. The mechanism causing apoptosis, however, is still controversial. Since the glucocorticoid receptor is a transcription factor, some of its target genes are expected to be implicated in apoptosis. In this study, using a GC-sensitive human pre-B leukemia cell line, Nalm-6, the *FK506 binding protein 51* (*FKBP5*) and *regulator of calcineurin 1* (*RCAN1*) genes were disrupted by homologous recombination, since the expression of both is up-regulated by GC in GC-sensitive but not in GC-resistant leukemic cell lines. While the disruption of *FKBP5* had a marginal effect on GC-induced apoptosis, that of *RCAN1* resulted in marked resistance to GC. In addition, overexpression of *RCAN1* rendered cells more sensitive to DEX. In *RCAN1*-disrupted cells, levels of some pro-apoptotic and anti-apoptotic Bcl-2 family proteins were decreased and increased, respectively. Finally, phosphorylation of cAMP-response element binding protein (CREB) and up-regulation of CREB target genes by GC were inhibited by *RCAN1* disruption, and treatment with a cAMP-inducing agent, forskolin, restored the sensitivity to GC in *RCAN1*-disrupted Nalm-6 cells. These findings suggest that up-regulation of *RCAN1* expression followed by activation of the CREB pathway is required in GC-induced apoptosis.

## Introduction

Glucocorticoids (GCs) have a wide variety of pharmacological effects such as immunosuppression, anti-allergy, and anti-inflammation, and are also used as chemotherapeutic agents for various leukemias, lymphomas, and multiple myelomas because of their ability to induce apoptosis and cell cycle arrest in lymphoid cells [1], [2]. GCs diffuse through the cell membrane into the cytoplasm and bind to the intracellular glucocorticoid receptor (GR) to exert their effects on target cells. Ligand-free GR is largely present in the cytoplasm as a large multi-protein complex which includes various chaperone proteins such as heat shock protein 90 (Hsp90) and tetratricopeptide repeat (TRP) proteins [3], [4]. The binding of GC triggers a conformational change in GR resulting in the dissociation of Hsp90 and GR translocates into the nucleus where it binds directly to a specific palindromic sequence, termed the glucocorticoid response element (GRE) as a dimer, resulting in the transactivation of various target genes [5]. Alternatively, GR interacts with other transcription factors such as AP-1, STAT-5, and NF-κB [6]–[8]. These interactions affect transcription levels of the target genes by modifying the actions of the GR partners.

Although GC-induced apoptosis is reported to require GR-mediated gene changes [9], the genes responsible for induction of apoptosis are not completely understood. We previously showed that 93 genes were transcriptionally up-regulated in a pre-B human leukemia cell line, 697, during GC-induced apoptosis using oligo-nucleotide microarrays. Importantly, some of these genes were up-regulated in a wide variety of other GC-sensitive pre-B leukemic cell lines such as Nalm-20, Nalm-27, and Nalm-6 [10]. Among them were *FK506 binding protein 51* (*FKBP5*) and *regulator of calcineurin 1* (*RCAN1*), considered candidates for genes that mediate GC-induced apoptosis in lymphoid cells for the following reasons. First, the activation of calcineurin protects T cells from GC-induced apoptosis [11]. Second, using a variety of pre-B leukemic cell lines, the degree of induction of these genes and of apoptosis were found to be closely correlated [12].

The FKBP51 protein encoded by *FKBP5* is a member of the highly conserved FKBP family. Also known as immunophilins, these proteins contain an FK506-binding domain that binds to immunosuppressive drugs such as FK506, rapamycin and cyclosporin A, and inhibit the serine/threonine phosphatase activity of calcineurin [13]. In addition, some tetratricopeptide repeat (TRP) domain-containing members of the FKBP family, such as FKBP52 and FKBP51, interact with GR through an Hsp90 dimer and modulate nuclear translocation of GR [14].

*RCAN1*, also known as *Down syndrome critical region 1* (*DSCR1*) or *modulatory calcineurin-interacting protein 1* (*MCIP1*), encodes two major protein isoforms of calcineurin inhibitors, *RCAN1–1* and *RCAN1–4*, via alternatively used first exons [15]. Expression of the *RCAN1* isoforms is differentially induced in response to various stress signals [16], [17]. We previously reported that a synthetic GC, dexamethasone (DEX), induced transcription of *RCAN1–1* but not *RCAN1–4* which is reported to be induced by calcium stress [12]. Both isoforms of RCAN1 modulate calcium-calcineurin-NFAT signaling by interacting with calcineurin via the C-terminus of RCAN1 [18], [19]. Phosphorylation of the RCAN1 protein at conserved serine residues was reported to alter its ability to bind with calcineurin. Unphosphorylated RCAN1 binds to calcineurin and inhibits its activity, while phosphorylated RCAN1 is incapable of binding to calcineurin, resulting in its increased activity [20]. Activation of calcineurin signaling is also suggested to confer resistance to GC in T cell-derived cells including lymphoma cell lines [20]. In addition, RCAN1 is suggested to attenuate NF-κB-mediated transactivation by stabilizing the inhibitory protein of NF-κB, IκBα, leading to impaired cell survival [21].

Therefore, we speculated that these two genes, *FKBP5* and *RCAN1*, play an important role in GC-mediated apoptosis. To clarify this role, we employed Nalm-6 cells and disrupted *FKBP5* and *RCAN1* alleles independently. Nalm-6, a human pre-B acute lymphoblastic leukemia cell line, is highly effective in gene-targeting experiments and also sensitive to GC [22].

**Figure 1:**
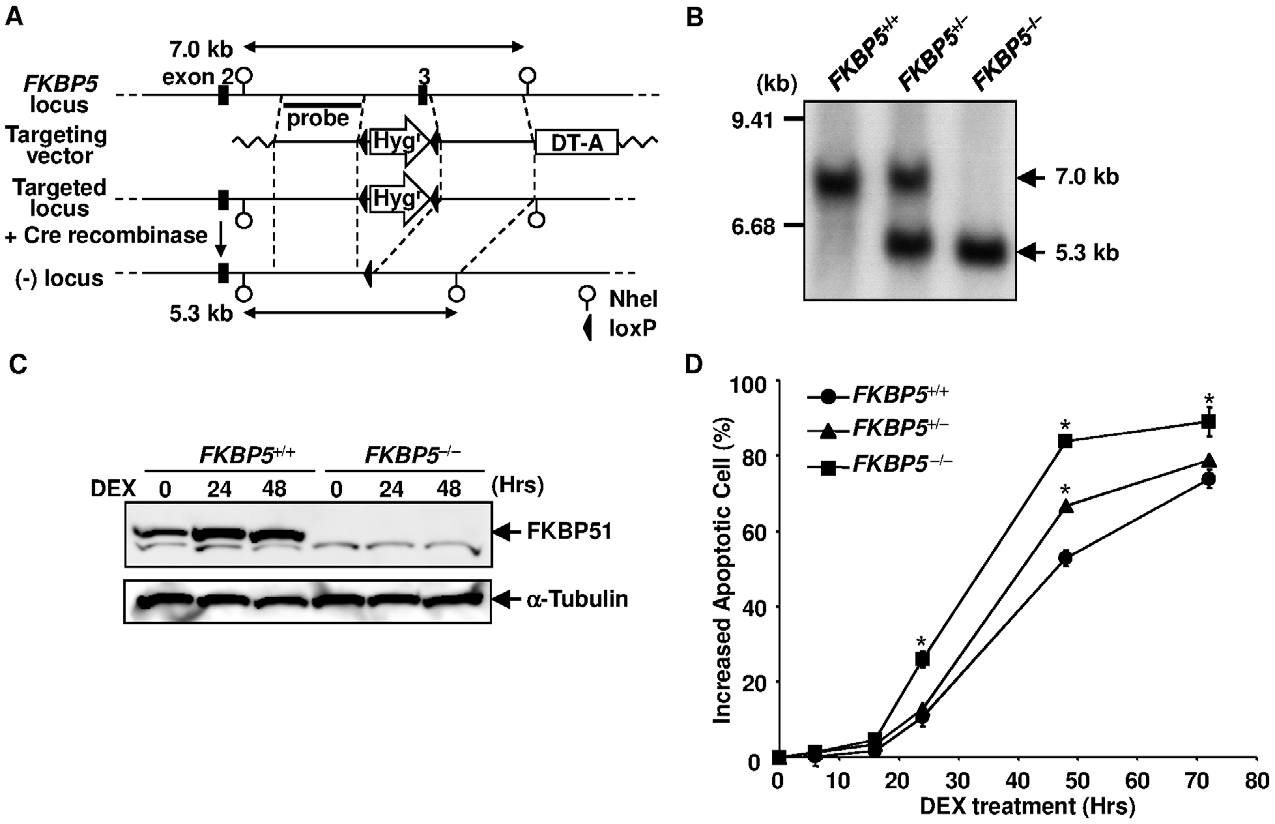
Targeted disruption of the human *FKBP5* locus. (**A**) Schematic representation of the *FKBP5* locus and the targeting vector. Exon 3 of the human *FKBP5* gene was replaced with a floxed *Hyg* ^r^ gene by gene targeting. The *Hyg* ^r^ gene was then removed by transfecting the Cre recombinase expression vector. (**B**) Southern blot analysis of the targeted clones. NheI-digested genomic DNA was analyzed. The probe used for the analysis is depicted in panel A. (**C**) Western blot analysis of FKBP51 expression. Cell lysate was extracted from DEX-treated cells at the indicated time points and subjected to Western blotting using anti-FKBP51 and anti-tubulin antibodies. (**D**) Time course of cell death induced by DEX. *FKBP5* ^+/+^, *FKBP5* ^+/−^, and *FKBP5* ^−/−^ cells were treated with 10^−6^ M of DEX. Apoptosis was assessed by flow cytometry using annexin V-PE at various time points. Error bars represent S.D. (*n = 3*). *, p<0.01 vs. *FKBP* ^+/+^.

**Figure 2:**
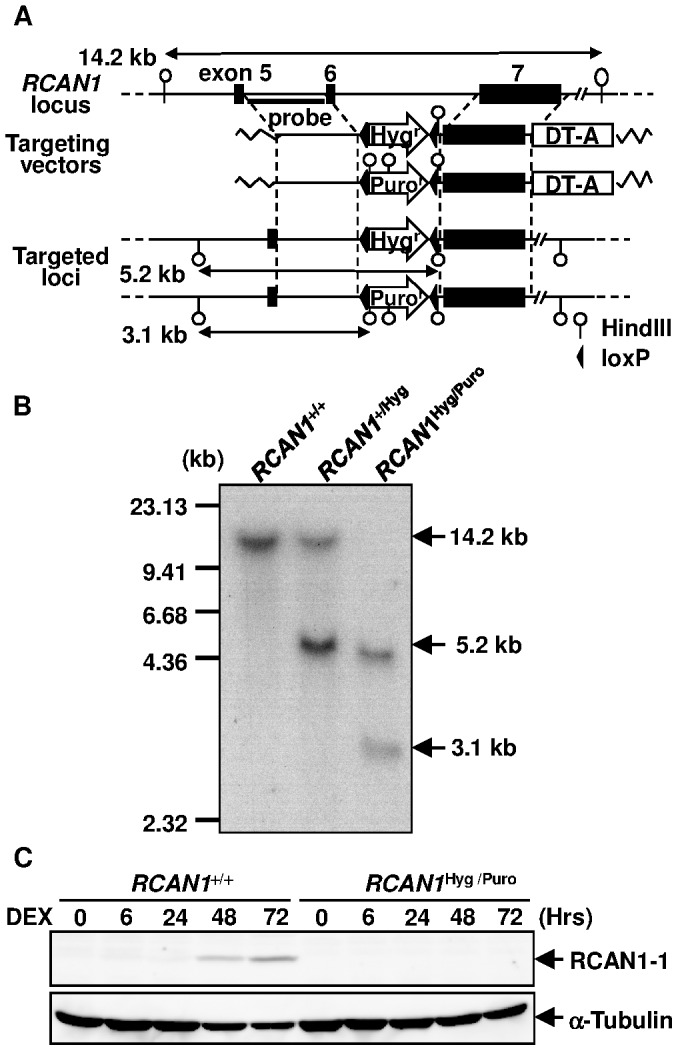
Targeted disruption of the human *RCAN1* locus. (**A**) Schematic representation of the *RCAN1* locus and the targeting vectors. The human *RCAN1* gene, located at 21q22, composed of 4 alternatively used first exons (exon 1–4) and 3 commonly used exons (exons 5–7). Exon 6 was replaced with a hygromycin- or puromycin-resistance gene by gene targeting. (**B**) Southern blot analysis of the targeted clones. HindIII-digested genomic DNA was analyzed. The probes used for the analysis are depicted in panel A. (**C**) Western blot analysis of RCAN1 expression. Cell lysate was extracted from DEX-treated cells at the indicated time points and subjected to Western blotting using anti-RCAN1 and anti-tubulin antibodies.

## Materials and Methods

### Cell Culture Conditions

The human pre B cell line, Nalm-6, which was obtained from Cell Resource Center for Biomedical Research (Tohoku University School of Medicine, Japan), and its derivatives were maintained in RPMI 1640 medium supplemented with 10% fetal calf serum, 50 U/ml penicillin, and 0.1 mg/ml streptomycin at 37°C in a humidified atmosphere containing 5% CO_2_ [23]. For some experiments, a recombinant human TRAIL and an anti-His_6_ cross linking antibody (R&D Systems, Minneapolis, MN) were added to the medium at a concentration of 10 ng/ml and 1 µg/ml, respectively.

### Targeting Constructs

The construction of targeting vectors was based on a simplified system [24]. In brief, for the human *RCAN1* gene, 1.9-kb and 2.1-kb genomic DNA fragments were amplified by Expand high fidelity PCR systems (F. Hoffmann-La Roche, Ltd., Basel, Switzerland) with Nalm-6 genomic DNA as a template and used as a 5′-arm and a 3′-arm, respectively. For the human *FKBP5* gene, 2.2-kb and 1.7-kb fragments were obtained and used as a 5′-arm and a 3′-arm, respectively. The primer sequences are listed in Table S1. A floxed hygromycin-resistance gene (*Hyg* ^r^) or puromycin-resistance gene (*Puro* ^r^) was inserted between the 5′- and 3′-arms on a plasmid carrying a diphtheria toxin A (DT-A) gene using MultiSite Gateway Technology® (Invitrogen, Carlsbad, CA), yielding the targeting vectors pRCAN1-Hyg, pRCAN1-Puro, and pFKBP5-Hyg. The entry clones containing *Hyg* ^r^ and *Puro* ^r^, pENTR lox-Hyg and pENTR lox-Puro, and destination vector containing DT-A, pDEST DTA-MLS, were kindly provided by Dr. Adachi (Yokohama City University, Yokohama, Japan) [24]. The targeting vectors were linearized by digestion with the restriction enzyme PmeI (New England Biolabs, Inc., Ipswich, MA), purified by phenol/chloroform extraction, and subjected to transfections.

**Figure 3:**
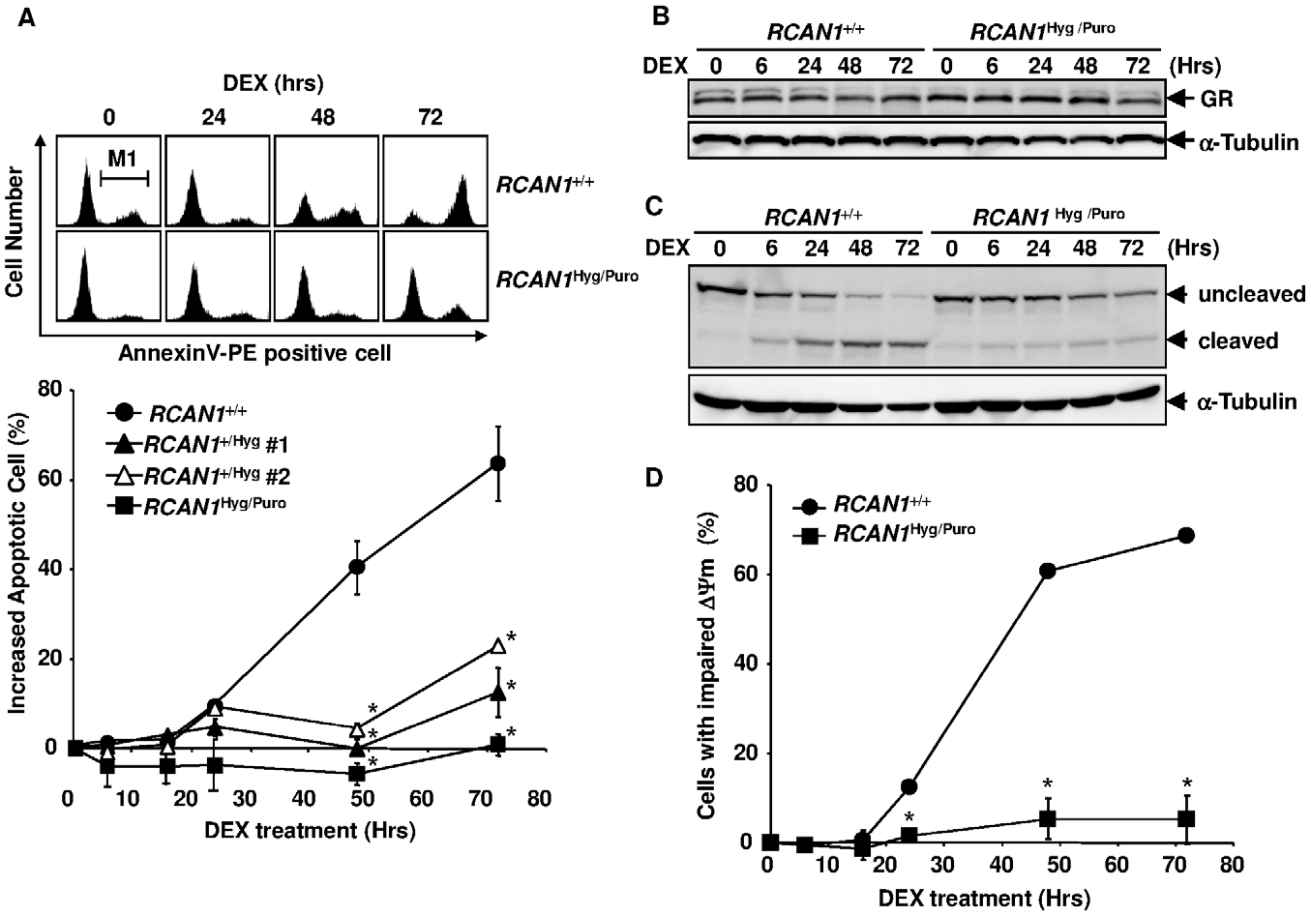
*RCAN1* disruption inhibited GC-induced apoptosis. (**A**) Time course of cell death induced by DEX. Wild-type Nalm-6 (*RCAN1* ^+/+^), *RCAN1* ^+/Hyg^, and *RCAN1* ^Hyg/Puro^ cells were treated with 10^−6^ M of DEX. Apoptosis was assessed by flow cytometry using annexin V-PE at various time points. The cell population gated by M1 was regarded as apoptotic. (**B**) Western blot analysis of GR expression. Cell lysate was extracted from DEX-treated cells at the indicated time points and subjected to Western blotting using anti-GR and anti-tubulin antibodies. (**C**) Proteolytic cleavage of PARP. Cell lysate was extracted from DEX-treated cells at the indicated time points and subjected to Western blotting using an anti-PARP and anti-tubulin antibodies. (**D**) *RCAN1* ^+/+^ and *RCAN1* ^Hyg/Puro^ cells were treated with 10^−6^ M DEX. The cells were harvested, stained with DiOC_6_(3), and subjected to flow cytometry at various time points. (A, D) Error bars represent S.D. (*n = 3*). *, p<0.01 vs. *RCAN1* ^+/+^.

### Generation of *FKBP5-* and *RCAN1-*knock-out Nalm-6 and RCAN1-overexpressing Nalm-6 Cells

Nalm-6 cells (4×10^6^) were electroporated with 4 µg of linearized targeting vector by using Nucleofector™ (Lonza, Basel, Switzerland) according to the manufacturer’s instructions. After 24 hours of incubation, the cells were replated into two 96-well plates with medium containing 0.4 mg/ml of hygromycin B (Wako Pure Chemical Industries, Osaka, Japan). After 3–4 weeks of incubation, hygromycin-resistant clones were isolated and genomic DNA was extracted from each clone using a QIAamp® DNA Mini Kit (Qiagen, Hilden, Germany). The primers used for PCR screening were as follows: RCAN1 5′-F and universal primer B for detecting 5′-arm homologous recombination, and RCAN1 3′-R and universal primer A for detecting 3′-arm homologous recombination. *RCAN1* ^+/Hyg^ cells were then electroporated with linearized pRCAN1-Puro and selected with 0.2 µg/ml of puromycin (Wako Pure Chemical Industries). PCR screening was performed using RCAN1 5′-F and universal primer A for the 5′-arm, and RCAN1 3′-R and universal primer B for the 3′-arm to identify *RCAN1* ^Hyg/Puro^ cells. Proper homologous recombination was further confirmed by Southern blotting. To generate cells overexpressing *RCAN1*, Nalm-6 cells were transfected with 4 µg of pHA-RCAN1 (kindly provided by Dr. de la Luna, Hospital Duran i Reynals) and selected with medium containing 0.7 mg/ml of G418 (Takara Bio Inc., Otsu, Japan). For the disruption of *FKBP5* in Nalm-6 cells, pFKBP5-Hyg was transfected and the hygromycin-resistant clone *FKBP5* ^+/Hyg^ was confirmed by PCR using FKBP5 5′-F and universal primer B for the 5′-arm, and FKBP5 3′-R and universal primer A for the 3′-arm. Then, *FKBP5* ^+/Hyg^ was transiently transfected with the Cre recombinase expressing vector, pEF-Cre (kindly provided by Dr. Miyado, National Center for Child Health and Development), to excise the hygromycin resistance gene (*FKBP5* ^+/−^). *FKBP5* ^+/−^ cells were further transfected with pFKBP5-Hyg and the resulting clone *FKBP5* ^−/Hy*g*^ was repeatedly transfected with pEF-Cre to generate *FKBP5* ^−/−^ cells.

### Southern Blotting

To confirm the disruption of *RCAN1* alleles, 8 µg of genomic DNA extracted from Nalm-6, *RCAN1* ^+/Hyg^, and *RCAN1* ^Hyg/Puro^ cells was digested with HindIII (New England Biolabs), resolved in 0.7% agarose gel by electrophoresis, and transferred onto a GeneScreen Plus® hybridization transfer membrane (PerkinElmer, Waltham, MA). The hybridization was carried out at 65°C for 16 hours using an α-[^32^P]-dATP-labeled PCR product amplified with primers RCAN1 5′-F2 and RCAN1 5′-R2 as a probe. After the hybridization, the blot was washed twice with 2×SSC, 0.1% SDS for 5 min each at room temperature, and twice with 0.2×SSC, 0.1% SDS for 15 min each at 65°C. The radioactive signal was visualized using a FLA2000 phosphorimager (Fujifilm, Tokyo, Japan). To confirm the disruption of *FKBP5* alleles, NheI (New England Biolabs) was used instead of HindIII and probed with an α-[^32^P]-dATP-labeled PCR product amplified with primers FKBP5 5′-F2 and FKBP5 5′-R2.

### Western Blotting

Immunoblot analysis was performed as described previously [25]. In brief, 30 µg of cell lysate was subjected to SDS–PAGE and transferred onto a nitrocellulose membrane. Anti-FKBP51 (Santa Cruz, Santa Cruz, CA), anti-RCAN1 (Santa Cruz), anti-hemagglutinin (HA) (F. Hoffmann-La Roche, Ltd.), anti-GR (Santa Cruz), anti-poly (ADP-ribose) polymerase (PARP) (Enzo Life Sciences, Farmingdale, NY), Anti-BAX (Medical & Biological Laboratories, Nagoya, Japan), anti-Bcl-2 (Cell Signaling Technology, Danvers, MA), anti-Bcl-xL (Cell Signaling Technology), anti-Bax (Cell Signaling Technology), anti-Bim (Cell Signaling Technology), anti-CREB (Cell Signaling Technology), anti-phosphorylated CREB (Ser133) (Cell Signaling Technology), and anti-Bak [26] antibodies were used as a primary antibodies. Horse radish peroxidase-conjugated anti-rat IgG, anti-rabbit IgG (Santa Cruz) and anti-mouse IgG (DAKO, Tokyo, Japan) were used as secondary antibodies. The proteins were visualized using enhanced chemiluminescence immunoblotting detection reagents (GE Healthcare, Buckinghamshire, England).

**Figure 4:**
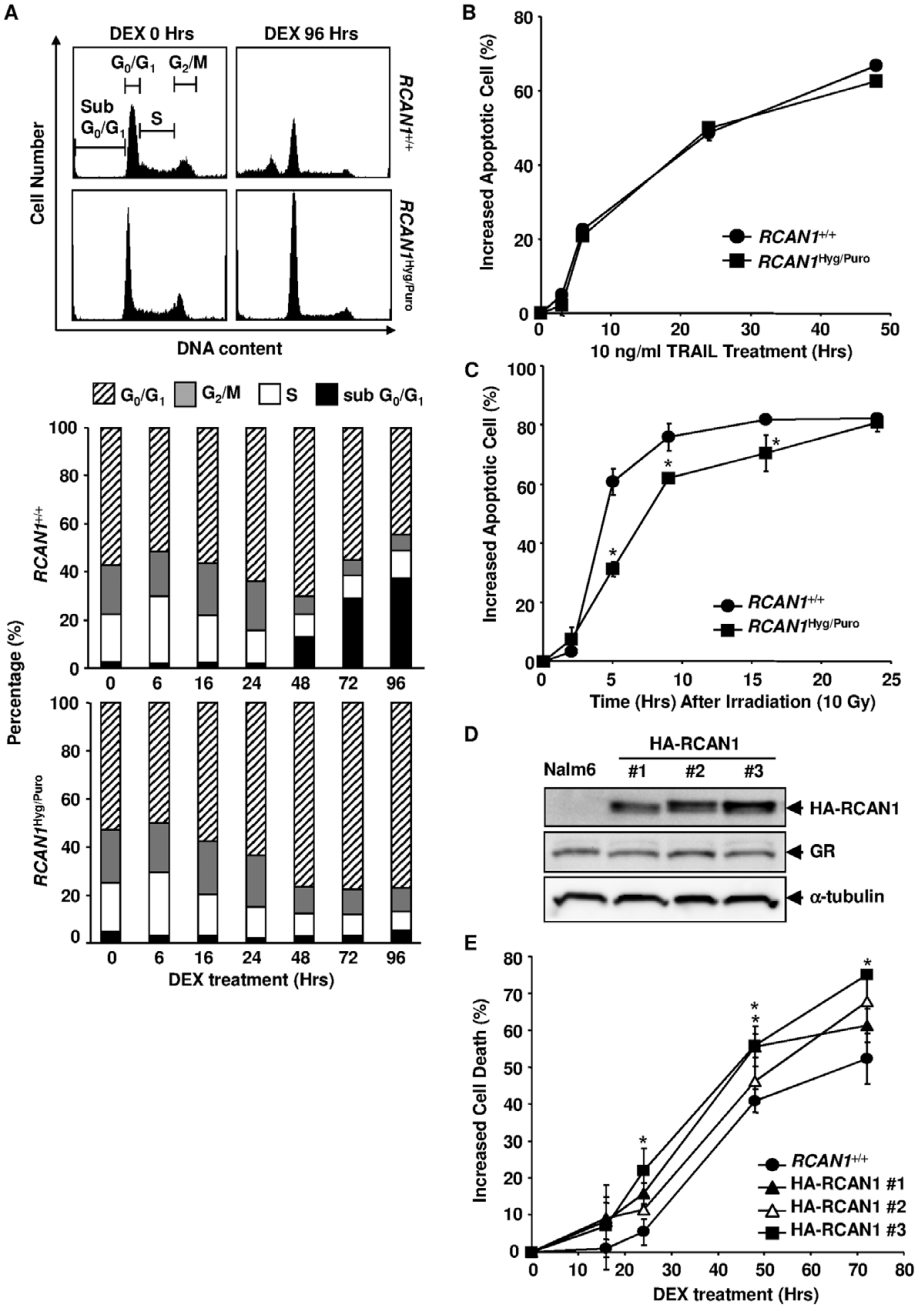
The effects of altered *RCAN1* expression on the cell cycle and apoptosis. (**A**) Flow cytometric profiles of DNA content after the GC treatment. Histograms of the DNA content are depicted at the top. Cells were gated as subG_0_/G_1_, G_0_/G_1_, S and G_2_/M. The percentage of cells in each population is represented at the bottom. The results are presented as the mean for three independent experiments. (**B, C**) Annexin V-bound apoptotic *RCAN1* ^+/+^ and *RCAN1* ^Hyg/Puro^ cells were counted flow cytometrically at various time points after treatment with 10 ng/ml of TRAIL and 1 µg/ml of cross linking anti-His_6_ antibody (**B**) or 10 Gy of irradiation (**C**). (**D**) Enforced expression of HA-tagged RCAN1. Nalm-6 cells were transfected with pHA-RCAN1. G418-resistant clones were subjected to Western blotting using an anti-HA, anti-GR and anti-tubulin antibodies. (**E**) *RCAN1* ^+/+^ and *RCAN1* ^Hyg/Puro^ cells were treated with 10^−6^M DEX and apoptosis was flow cytometrically assessed by annexin V-binding at various time points. (B, C and E) Error bars represent S.D. (*n = 3*). *, p<0.05 vs. *RCAN1* ^+/+^.

### Flow Cytometry

Cells (5×10^5^/ml) were treated with 1 µM DEX. At various time points, the percentage of apoptotic cells, the relative DNA content of the cells, and mitochondrial membrane potential (ΔΨm) were determined by flow cytometry using a FACScan (Becton, Dickinson and Company, Franklin Lakes, NJ). To analyze cell viability, PI was added to the culture at 40 µg/ml and the cells subjected to flow cytometry. To measure apoptosis, the cells were collected and washed twice with ice-cold PBS. Then, they were resuspended in 100 µl of binding buffer at a concentration of 1×10^6^ cells/ml, 5 µl each of Annexin-V-PE (Becton, Dickinson and Company) and 50 µg/ml 7-AAD (Becton, Dickinson and Company) were added, and incubation was continued for 15 min at room temperature in the dark. After 400 µl of binding buffer was added, the cells were subjected to flow cytometry. Assays for the relative DNA content of the cells and ΔΨm were performed as described previously [27]. In brief, relative DNA content was determined by staining the cells with a PI-containing solution (50 µg/ml PI, 0.1% triton-X 100, 0.1 mM EDTA, and 50 µg/ml RNase A in PBS [pH 7.4]). The ΔΨm was determined by staining the cells with 40 nM DiOC_6_(3) (Invitrogen).

### Quantitative RT-PCR

Five micrograms of total RNA extracted using TRIzol reagent (Invitrogen) was reverse-transcribed by the Superscript First-Strand Synthesis System in a final volume of 20 µl (Invitrogen) according to the manufacturer’s instructions. The resulting cDNA was used as a template for quantitative PCR performed with SsoFast EvaGreen Supermix and the CFX96 Real Time System (Bio-Rad Laboratories, Hercules, CA). Primers used for the analysis are listed in Table S1.

**Figure 5:**
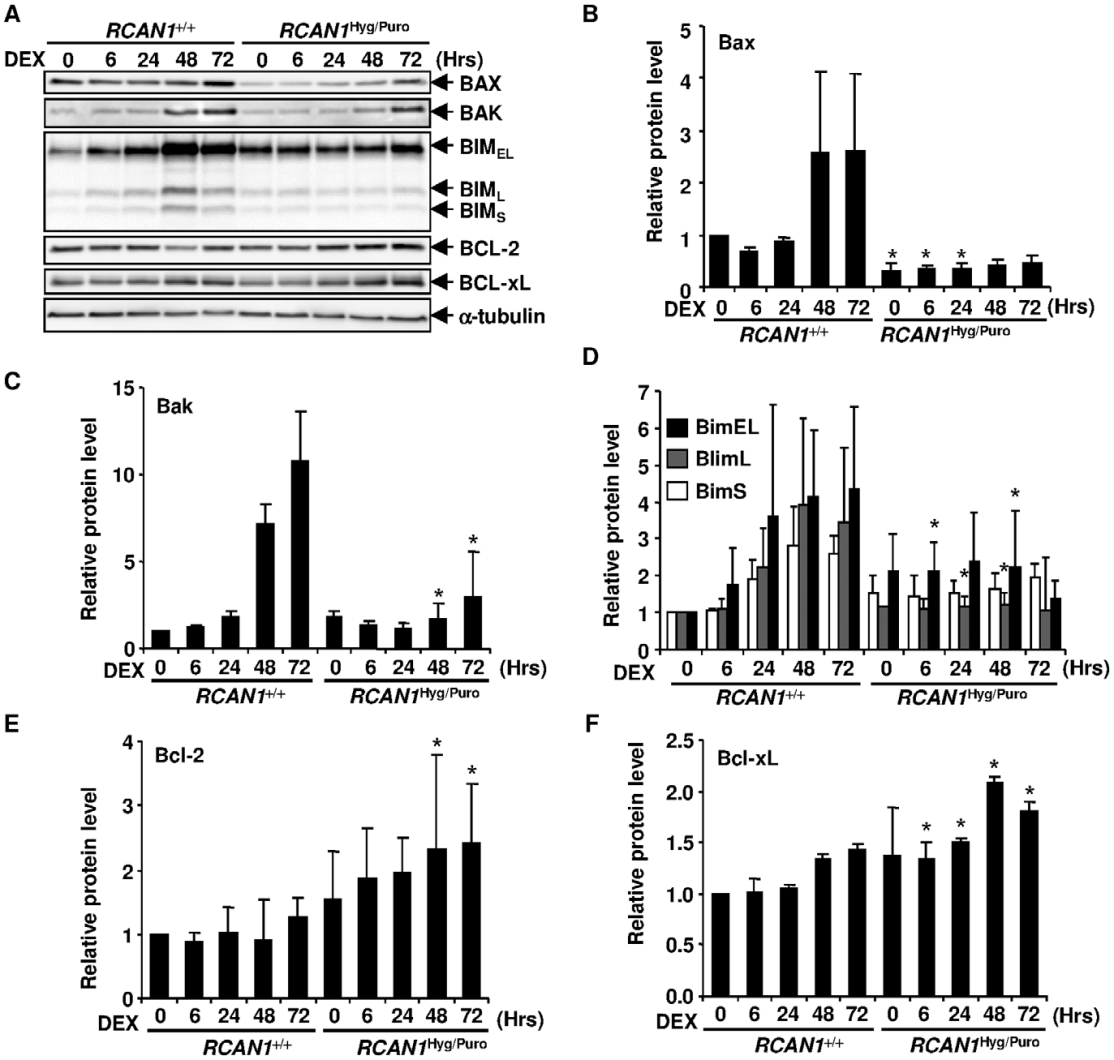
*RCAN1* disruption changed expression levels of Bcl2 family proteins. (**A**) 10^−6^ M DEX-treated *RCAN1* ^+/+^ and *RCAN1* ^Hyg/Puro^ cells were harvested at indicated time points and cell lysate was subjected to Western blotting with anti-Bax, -Bak, -Bim, -Bcl-2 and -Bcl-xL antibodies. Anti-α-tubulin antibody was used for the loading control. (**B–F**) The signal intensity of BAX (**B**), BAK (**C**), each BIM isoform (**D**), Bcl-2 (**E**) and Bcl-xL (**F**) obtained in (**A**) was quantified and normalized to that of α-tubulin. *, p<0.05 vs. *RCAN1* ^+/+^ at the same time point.

## Results

### Disruption of the Human *FKBP5* Locus did not Affect GC Sensitivity

The transfection of Nalm-6 cells with pFKBP5-Hyg and subsequent selection with hygromycin B yielded the *FKBP5* ^+/Hyg^ clone (Figure 1A). The transfection of *FKBP5* ^+/Hyg^ cells with pEF-Cre, then pFKBP5-Hyg, and finally pEF-Cre again, produced the *FKBP5* ^−/−^ clone. The disruption of *FKBP5* was confirmed by Southern blotting (Figure 1B) and Western blotting (Figure 1C). To determine whether the disruption alters the sensitivity to GC, we investigated the time course of cell death of wild-type and *FKBP5* ^−/−^ cells treated with 1 µM DEX. The percentage of annexin V-positive apoptotic cells among wild-type Nalm-6 cells started to increase at 24 hours after the DEX treatment and 40% of the cells were positive at 48 hours, while 63% of *FKBP5* ^−/−^ cells were apoptotic at this time point (Figure 1D). Therefore, unexpectedly, the disruption of the *FKBP5* gene resulted in a modest but significant enhancement of GC-induced apoptosis in Nalm-6 cells. This result was confirmed when cell viability was determined by propidium iodide (PI) exclusion (Figure S1A).

**Figure 6:**
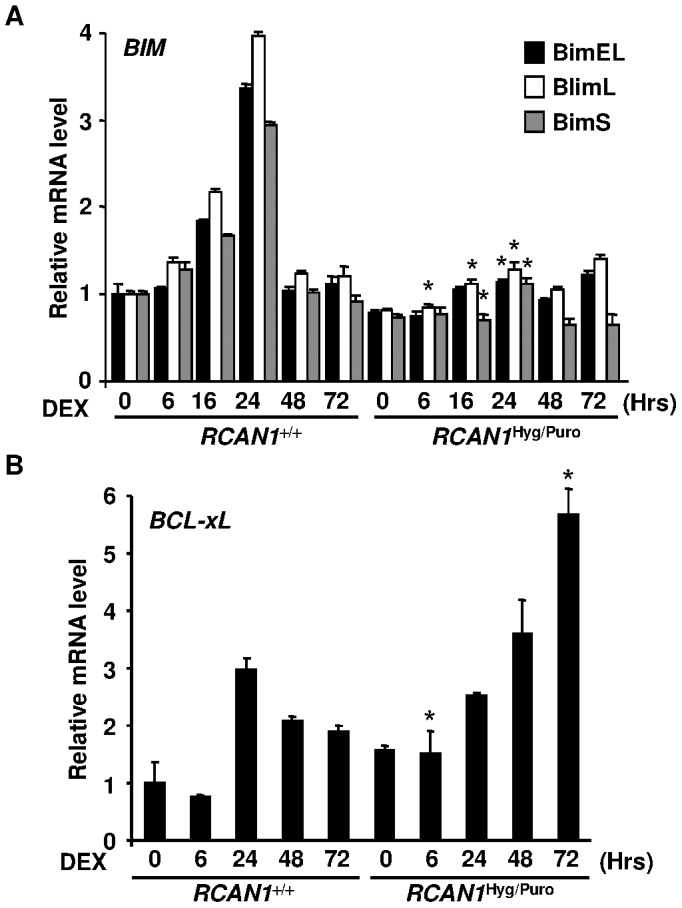
*RCAN1* disruption changed expression levels of *BIM* and *Bxl-xL* mRNAs. Total RNA was extracted from *RCAN1* ^+/+^ and *RCAN1* ^Hyg/Puro^ cells, treated with 10^−6^ M DEX for the period indicated, and subjected to quantitative real-time RT-PCR using primer sets hybridizing to each *BIM* isoform (**A**) (*, p<0.01 vs. *RCAN1* ^+/+^ at the same time point and the same isoform) and *Bcl-xL* (**B**) (*, p<0.05 vs. *RCAN1* ^+/+^ at the same time point). Each expression was normalized to that of *GAPDH.* Error bars represent S.D. (*n = 3*).

### GC-induced Apoptosis is Markedly Inhibited in *RCAN1* ^Hyg/Puro^ Cells

The transfection of Nalm-6 cells with pRCAN1-Hyg and subsequent selection with hygromycin yielded the *RCAN1* ^+/Hyg^ clone (Figure 2A). We then transfected the *RCAN1* ^+/Hyg^ cells with pRCAN1-Puro and selected cells with puromycin to establish the *RCAN1* ^Hyg/Puro^ clone. The disruption of *RCAN1* was confirmed by Southern blotting (Figure 2B) and Western blotting (Figure 2C). In contrast to *FKBP5* ^−/−^ cells, annexin V-positive apoptotic cells increased by only 22.8% after the DEX treatment for 72 hours in heterozygous knock out cells, *RCAN1* ^+/Hyg^ cells (Figure 3A). Apoptosis was almost completely inhibited in homozygous knock out cells, *RCAN1* ^Hyg/Puro^ cells (0.7% at the same time point). Even after 144 hours, only 8.6% of the *RCAN1* ^Hyg/Puro^ cells were annexin V-positive (71.8% of *RCAN1* ^+/+^ cells at this time point, data not shown). This result was confirmed when cell viability was determined by PI exclusion (Figure S1B). GC-induced cell death is known to be mediated by GR and, therefore, the levels of GR expression are important for GC-induced apoptosis. However, GR expression levels in *RCAN1* ^+/+^ cells were comparable to *RCAN1* ^Hyg/Puro^ cells (Figure 3B). The cleavage of poly (ADP-ribose) polymerase (PARP), a well-known substrate of caspase 3, observed in wild-type Nalm-6 cells after the treatment with DEX was also markedly inhibited in *RCAN1* ^Hyg/Puro^ cells (Figure 3C).

### *RCAN1* Disruption Inhibits the Loss of Mitochondrial Membrane Potential Induced by GC

The apoptotic pathway is divided into intrinsic (mitochondrial-dependent) and extrinsic (mitochondrial-independent) arms [28]. GC-mediated apoptosis is mediated via the former because GC-induced cell death and loss of mitochondrial membrane potential (ΔΨm) were completely inhibited by the overexpression of the mitochondrial protein, Bcl-2 [27], [29]. We next investigated whether the loss of ΔΨm was inhibited by *RCAN1* disruption in Nalm-6 cells. The percentage of wild-type Nalm-6 cells with loss of ΔΨm started to increase 24 hours after DEX treatment and reached 68.8% at 72 hours (Figure 3D). In contrast, this increase in the population with impaired ΔΨm was almost completely diminished among *RCAN1*-disrupted cells (5.2% at 72 hours), suggesting that *RCAN1* functions upstream of mitochondria.

**Figure 7:**
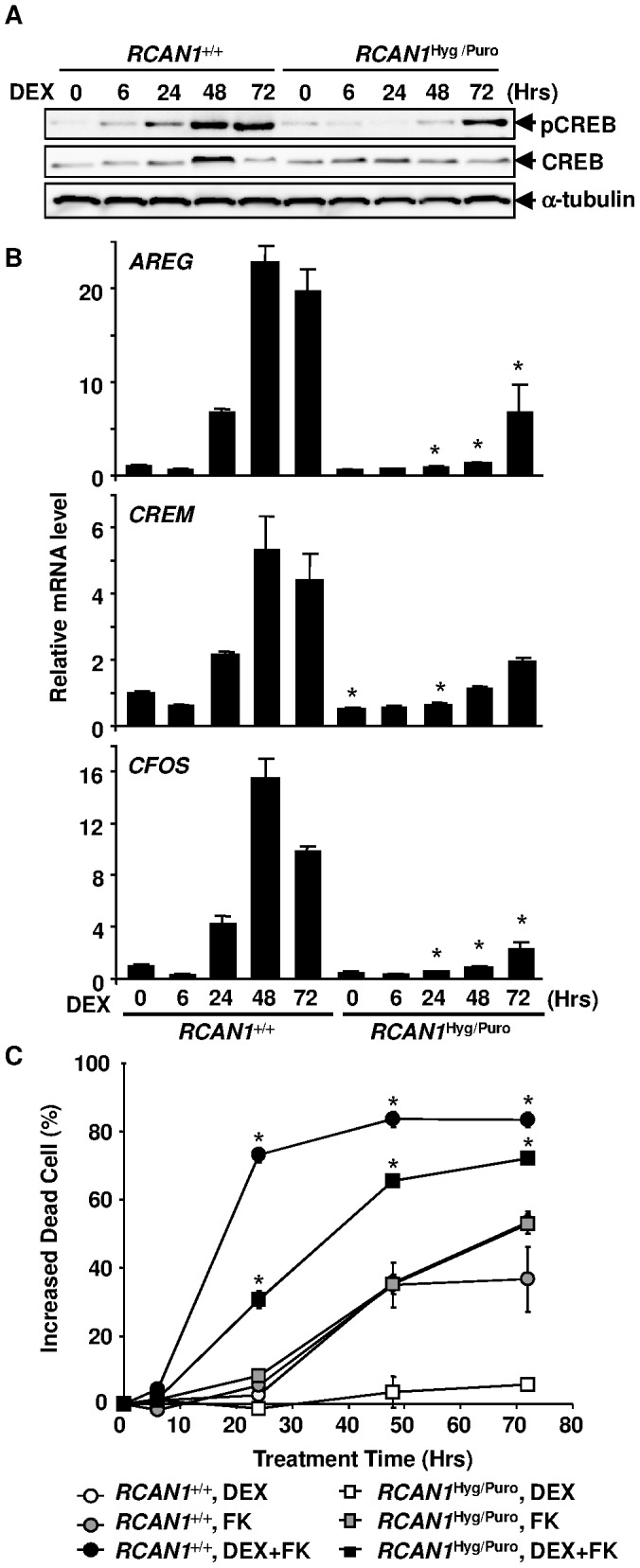
GC activates CREB in Nalm-6 cells. (**A**) Cell lysate obtained from DEX-treated *RCAN1* ^+/+^ and *RCAN1* ^Hyg/Puro^ cells was subjected to immunoblotting using anti-CREB and anti-pCREB (Ser133) antibodies. (**B**) RNA prepared from *RCAN1* ^+/+^ and *RCAN1* ^Hyg/Puro^ cells treated with 10^−6^ M DEX was subjected to quantitative RT-PCR using primer sets that hybridize to CREB target genes, *AREG*, *CREM*, and *CFOS*. *, p<0.01 vs. *RCAN1* ^+/+^ at the same time point. (**C**) *RCAN1* ^+/+^ and *RCAN1* ^Hyg/Puro^ cells pretreated with 10 µM forskolin (FK) for 1 hour were subsequently treated with 10^−6^ M DEX and subjected to flow cytometry. Dead cells were assessed by PI exclusion. Circles and squares indicate *RCAN1* ^+/+^ and *RCAN1* ^Hyg/Puro^ cells, respectively. Open, shaded, and closed symbols indicate the presence of DEX, forskolin, and DEX plus forskolin, respectively. *, p<0.05 vs. the same clone without FK treatment. (B, C) Error bars represent S.D. (*n = 3*).

### *RCAN1* Disruption does not Block Cell Cycle Arrest Evoked by DEX

GC is known to induce cell cycle arrest as well as apoptosis in certain lymphoid cells [30]. Thus, we next investigated whether the disruption of *RCAN1* affected the cell cycle as well. In accordance with annexin V staining, the sub G_0_/G_1_ population, indicative of apoptotic cells, increased in *RCAN1* ^+/+^ cells but not in *RCAN1* ^Hyg/Puro^ cells (Figure 4A). In contrast, both *RCAN1* ^+/+^ and *RCAN1* ^Hyg/Puro^ cells exhibited a clear decrease in the percentage of S and G2/M phase cells, accompanied by a relative increase in the G_0_/G_1_ population after the DEX treatment, indicating that *RCAN1* is unlikely to mediate GC-induced cell cycle arrest.

### *RCAN1* Disruption Predominantly Inhibits GC-induced Apoptosis

We subsequently investigated whether the effect of *RCAN1* disruption on apoptosis is GC-specific. TRAIL-induced apoptosis is representative of the extrinsic pathways. While DNA-damage-induced apoptosis is mediated by an intrinsic pathway, it is distinct from GC-induced apoptosis because thymocytes in p53 null mice are markedly resistant to γ-irradiation but as sensitive as wild-type thymocytes to GC-induced apoptosis [31]. *RCAN1* disruption had no effect on TRAIL-induced apoptosis (Figure 4B), but partially inhibited γ-irradiation-induced apoptosis (Figure 4C), indicating that the effect of the disruption is specific to GC-induced apoptosis, although some overlap with other mitochondria-dependent apoptosis may exist.

### The Effect of Overexpression of RCAN1 on GC-induced Apoptosis

If *RCAN1* functions as a mediator in GC-induced apoptosis, its overexpression is expected to have a facilitative effect on apoptosis. To address this issue, we established 3 clones of Nalm-6 stably expressing the RCAN1 protein by transfecting a hemagglutinin (HA)-tagged *RCAN1* expression vector (Figure 4D). The levels of GR expression were not impacted during the process of transfection and selection (Figure 4D). All the clones were more sensitive to DEX than the parental Nalm-6 cells (Figure 4E). These results further indicate that *RCAN1* plays an important role in GC-induced apoptosis.

### *RCAN1* Disruption Alters Expression Levels of Bcl-2 Family Proteins

We next investigated the levels of the Bcl-2 family proteins regulating the mitochondria-dependent apoptosis. In wild-type Nalm-6 cells, pro-apoptotic Bax, Bak and Bim expression was up-regulated (Figure 5A–D). These changes were inhibited, at least in part, in the *RCAN1*-disrupted Nalm-6 cells. Conversely, the expression of the anti-apoptotic Bcl-2 family proteins, Bcl-2 and Bcl-xL, was up-regulated up to two fold in *RCAN1*-disrupted Nalm-6 cells (Figure 5A, 5E and 5F). To examine whether these Bcl-2 family proteins were transcriptionally regulated or not, we performed a quantitative real-time RT-PCR analysis. Transcription of *BIM* in Nalm-6 cells and *Bcl-xL* in *RCAN1*-disrupted cells was up-regulated upon DEX treatment (Figure 6A and 6B). However, other Bcl-2 family genes, *BAX*, *BAK* and *BCL2*, were not changed significantly (Figure S2), suggesting that the protein levels of Bax, Bak and Bcl-2 were post-transcriptionally regulated. Taken together, it was suggested that the resistance to loss of ΔΨm in GC-treated *RCAN1*-disrupted Nalm-6 cells is explained by the changes in expression levels of Bcl-2 family proteins.

### CREB was Activated during DEX-induced Apoptosis

cAMP response element-binding protein (CREB), a well-studied transcriptional regulator, exerts its ability to regulate the cAMP response element-mediated transcription of target genes, such as *NR2A*, *AREG*, *CREM* and *CFOS*, in response to cAMP through phosphorylation at Ser133 by a cAMP-mediated protein kinase or Ca^2+/^calmodulin-dependent protein kinase [32]–[36]. It has been reported that the treatment of human B-precursor cells with cAMP and overexpression of the CREB protein in human amnion cells and CHO cells, induce apoptosis [37], [38]. Recently, overexpression of the *RCAN1* protein in the rat adrenal pheochromocytoma cell line, PC12, was reported to enhance the phosphorylation of CREB induced by an adenylate cyclase activator, forskolin [39]. Therefore, we next investigated the activation of the CREB pathway in *RCAN1*-disrupted Nalm-6 cells during DEX treatment. Phosphorylation of the Ser133 residue of CREB induced by DEX in *RCAN1* ^+/+^ cells was inhibited in *RCAN1* ^Hyg/Puro^ cells at least in part (Figure 7A). In accordance with this, CREB target genes, *AREG*, *CREM* and *CFOS*, were transcriptionally up-regulated during DEX treatment in *RCAN1* ^+/+^ cells but not in *RCAN1* ^Hyg/Puro^ cells (Figure 7B). These results indicate that the induction of *RCAN1* expression by DEX-treatment was followed by CREB activation. This prompted us to investigate whether the enforced phosphorylation of CREB by forskolin induces apoptosis in *RCAN1*-disrupted cells (Figure 7C). Indeed, forskolin induced apoptosis in *RCAN1* ^+/+^ and *RCAN1* ^Hyg/Puro^ cells to the same degree. Apoptosis was significantly enhanced by forskolin in the presence of DEX compared with that induced by forskolin or DEX alone, indicating that the phosphorylation of CREB mediates GC-induced apoptosis.

## Discussion

In this study, we knocked out *FKBP5* and *RCAN1* alleles independently in a human pre-B cell lymphoma cell line, Nalm-6, by gene targeting. The knock down of gene expression by RNA interference is frequently used to investigate gene function. However, the degree of knock-down is dependent on the RNA sequence and complete elimination of the target gene expression is impossible, and small interfering RNA sometimes has an off-target effect that reduces the expression of non-targeted genes [40]. Moreover, it is generally difficult to achieve high transfection efficiency in hematopoietic cells especially of B-cell origin. Therefore, we employed a targeted gene disruption strategy.

The disruption of *FKBP5* alleles in Nalm-6 resulted in increased sensitivity to DEX (69.7% of *FKBP5* ^+/+^ cells were dead versus 88.7% of *FKBP5* ^−/−^ cells at 72 hours) rather than inhibition of apoptosis. Other than functioning as a calcineurin inhibitor, the large immunophilin FKBP51 (encoded by *FKBP5*) is known to delay the nuclear translocation of GR [41], [42]. This may explain the unexpected effect of *FKBP5* disruption on GC-induced apoptosis.

In contrast, the disruption of *RCAN1* alleles significantly reduced sensitivity to DEX. What is the mechanism by which the induction of *RCAN1* expression causes apoptosis? During GC-induced apoptosis, CREB was phosphorylated in an *RCAN1*-dependent manner, consistent with a report that *RCAN1* increased the phosphorylation of CREB in rat PC12 cells [39]. The phosphorylation of CREB by *RCAN1* also depended on the inhibition of calcineurin in PC12 cells. However, in our study, inhibition of calcineurin by FK506 or cyclosporine A (CsA) did not induce apoptosis or enhance GC-mediated apoptosis in spite of the abundant expression of calcineurin in Nalm-6 cells (Figure S3). Therefore, the dependency on calcineurin of *RCAN1*-mediated CREB activation seems to be cell type-specific and, in Nalm-6 cells, *RCAN1* is likely to induce CREB activation through binding to another molecule or crosstalk with other signaling pathways.

We also demonstrated that levels of some of the Bcl-2 family proteins were altered by the disruption of the *RCAN1* alleles. Consistent with our results, the expression of *RCAN1–1* and *Bim* were up-regulated in CEM-C7–14, a DEX-sensitive human acute T-cell lymphoma cell line, but not in CEM-C1–15, a DEX-resistant sister clone of CEM-C7–14 [43], [44]. Although no complete CRE site was present in the promoter region of *Bim*, four half-CRE sites were found [45]. Moreover, the transcription of *Bim* was induced when human acute myeloma cells, IPC-81 cells, were treated with a protein kinase A (PKA)-specific activator, N^6^-MB-cAMP, in the presence of a protein synthesis inhibitor, cycloheximide, indicating that *Bim* is a direct target of CREB [46]. In contrast to the induction of *Bim* expression, that of *Bcl-xL* expression detected in *RCAN1*-disrupted Nalm-6 cells was not observed in wild-type Nalm-6 cells. *RCAN1* is reported to increase the stability of the IκBα protein via a calcineurin-independent mechanism leading to the decreased expression of NF-κB target genes such as *cylooxygenase-2* [21]. Since *Bcl-xL* is an NF-κB target gene [47], this may explain the mechanism of *Bcl-xL* suppression by *RCAN1*. PKA is reported to hyperphosphorylate Bcl-2 leading to a proteasome-mediated degradation in breast cancer cell lines [48]. This may explain post-transcriptional regulation of Bcl-2 by CREB. Altered expression patterns of Bcl-2 family proteins are consistent with the inhibition of GC-induced ΔΨm decrease in *RCAN1* knockout cells, since Bcl-2 family proteins regulate the mitochondrial apoptotic pathway.

Two recent studies reported a correlation between RCAN1 expression and sensitivity to GC in rat primary neurons [43], [49]. In the present study, by eliminating functional alleles of *RCAN1* in GC-sensitive Nalm-6 cells, we unequivocally demonstrated that the *RCAN1* gene is an important mediator of GC-induced apoptosis in leukemic cells.

## Supporting Information

Figure S1 **Time course of DEX-induced cell death assessed by the uptake of PI.** FKBP5- (**A**) and RCAN1- (**B**) disrupted cells were treated with 10^−6^ M of DEX. At the indicated time points, PI was added to the culture at 40 µg/ml and the cells were subjected to flow cytometry. Error bars represent S.D. (n = 3). *, p<0.01 vs. *FKBP5* ^+/+^ (A) and *RCAN1* ^+/+^ (B).
(PDF)

Figure S2 **Quantitative real-time RT-PCR analysis of Bcl-2 family genes.** Total RNA was extracted from *RCAN1* ^+/+^ and *RCAN1* ^Hyg/Puro^ cells, treated with 10^−6^ M DEX for the period indicated, and subjected to quantitative real-time RT-PCR using primer sets hybridizing to *BCL2*, *BAX* and *BAK*. Each expression was normalized to that of *GAPDH.* Error bars represent S.D. (*n = 3*).
(PDF)

Figure S3 **The effect of calcineurin inhibitors on GC-induced apoptosis in Nalm-6.** (**A**) One hour before DEX treatment, *RCAN1* ^+/+^ and *RCAN1* ^Hyg/Puro^ cells were pre-treated with 50 ng/ml cyclosporin A (CsA) and/or 50 nM FK506. At indicated time points, the cells were stained with Annexin V-PE and subjected to flow cytometric analysis. Error bars represent S. D. (*n = 3*). (**B**) CnA expression in human T-cell and B-cell lines. Total protein (30 µg) extracted from each cell line was subjected to immunoblotting and probed with anti-CnA and anti-tubulin antibodies.
(PDF)

Table S1 **The sequence of the oligonucleotide primers used in this study.**
(XLS)
