# Supplementary figures and images for: RCAN1 Is an Important Mediator of Glucocorticoid-Induced Apoptosis in Human Leukemic Cells
Source: PLoS One. 2012 Nov 21;7(11):e49926. doi: 10.1371/journal.pone.0049926 (PMC3503877; doi:10.1371/journal.pone.0049926)

Figure. S1

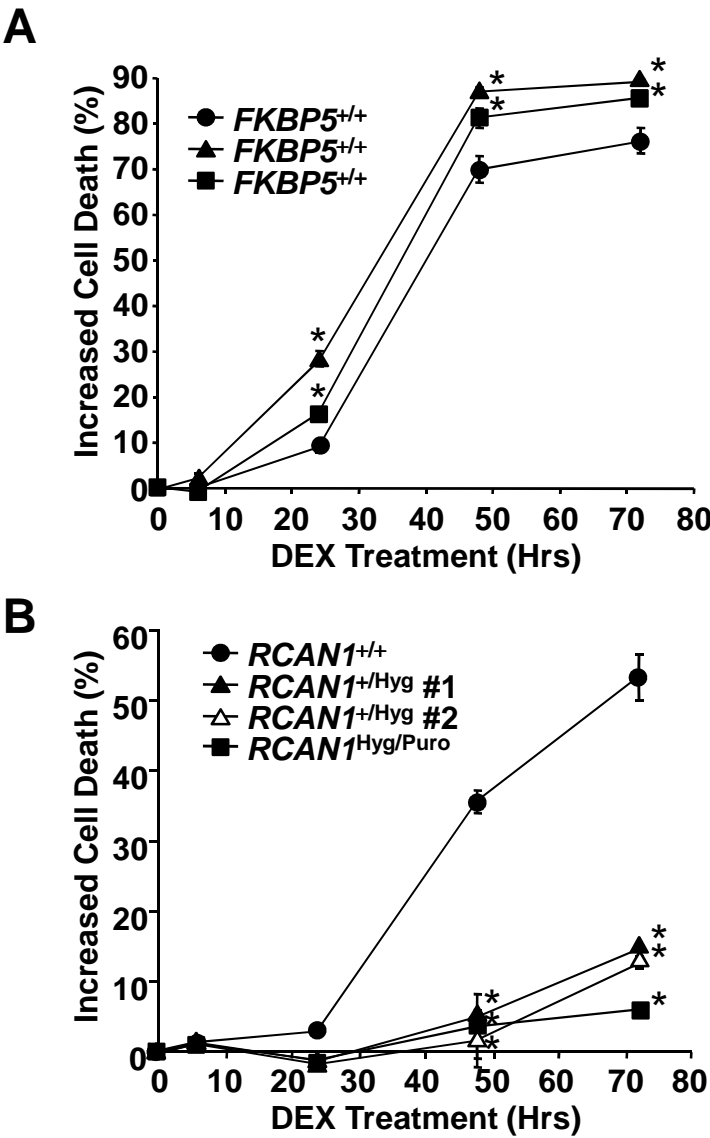

Supplement: Figure S1 — Time course of DEX-induced cell death assessed by the uptake of PI. FKBP5- (A) and RCAN1- (B) disrupted cells were treated with 10−6 M of DEX. At the indicated time points, PI was added to the culture at 40 µg/ml and the cells were subjected to flow cytometry. Error bars represent S.D. (n = 3). *, p<0.01 vs. FKBP5 +/+ (A) and RCAN1 +/+ (B). (PDF) [file pone.0049926.s001.pdf]

Figure. S2

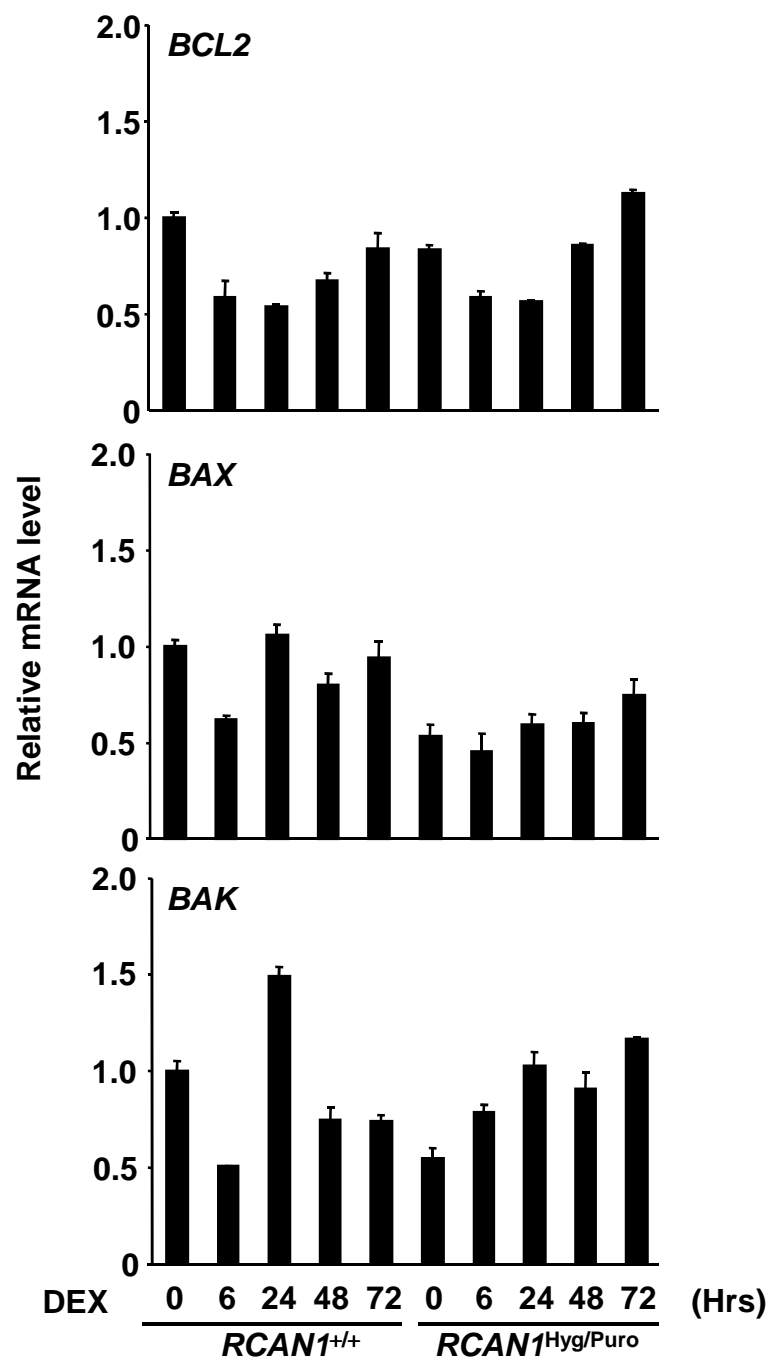

Supplement: Figure S2 — Quantitative real-time RT-PCR analysis of Bcl-2 family genes. Total RNA was extracted from RCAN1 +/+ and RCAN1 Hyg/Puro cells, treated with 10−6 M DEX for the period indicated, and subjected to quantitative real-time RT-PCR using primer sets hybridizing to BCL2, BAX and BAK. Each expression was normalized to that of GAPDH. Error bars represent S.D. (n = 3). (PDF) [file pone.0049926.s002.pdf]

Figure. S3

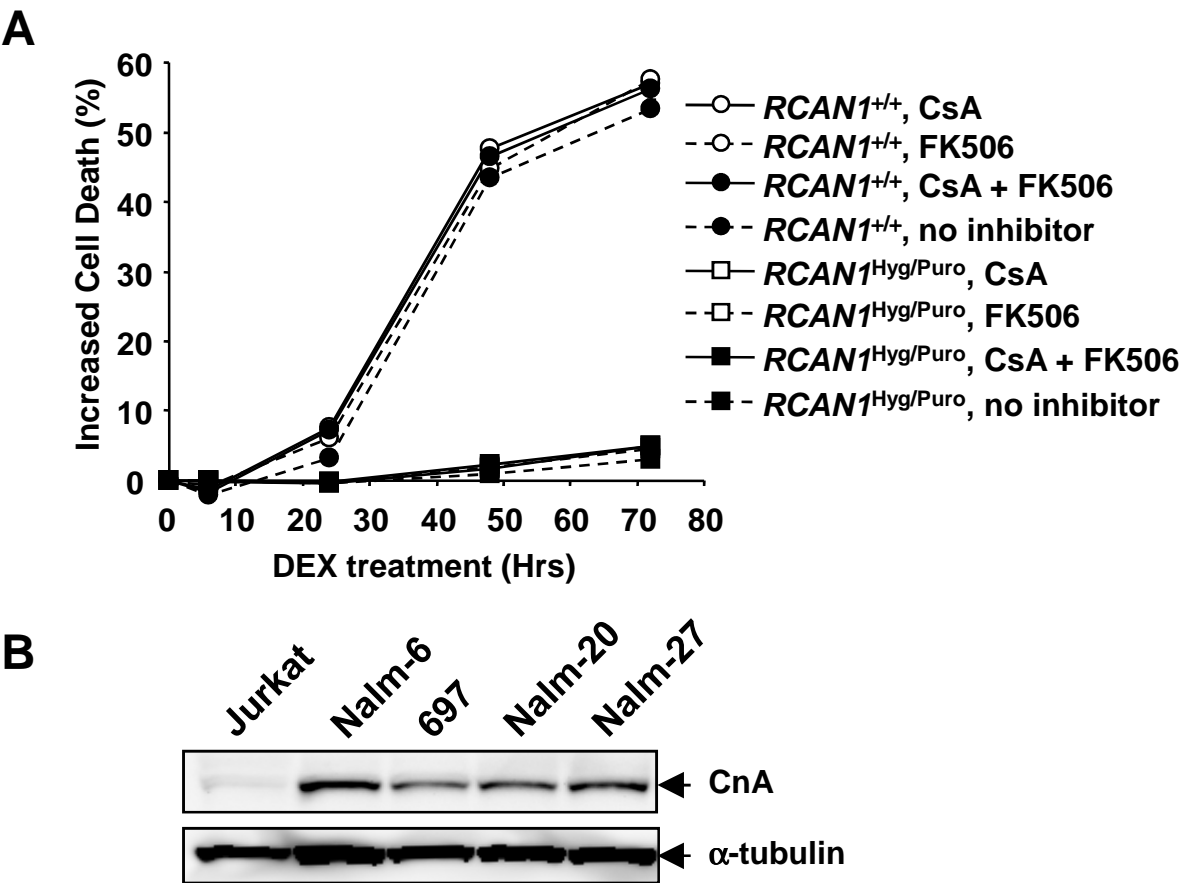

Supplement: Figure S3 — The effect of calcineurin inhibitors on GC-induced apoptosis in Nalm-6. (A) One hour before DEX treatment, RCAN1 +/+ and RCAN1 Hyg/Puro cells were pre-treated with 50 ng/ml cyclosporin A (CsA) and/or 50 nM FK506. At indicated time points, the cells were stained with Annexin V-PE and subjected to flow cytometric analysis. Error bars represent S. D. (n = 3). (B) CnA expression in human T-cell and B-cell lines. Total protein (30 µg) extracted from each cell line was subjected to immunoblotting and probed with anti-CnA and anti-tubulin antibodies. (PDF) [file pone.0049926.s003.pdf]
